# Utility and pitfalls of the electrocardiogram in the evaluation of cardiac amyloidosis

**DOI:** 10.1111/anec.12967

**Published:** 2022-05-14

**Authors:** Perryn Lin Fei Ng, Yoke Ching Lim, Lauren Kay Mance Evangelista, Raymond Ching Chiew Wong, Ping Chai, Ching Hui Sia, Hoi Yin Loi, Tiong Cheng Yeo, Weiqin Lin

**Affiliations:** ^1^ Department of Cardiology National University Heart Centre Singapore Singapore; ^2^ Yong Loo Lin School of Medicine National University of Singapore Singapore Singapore; ^3^ Department of Diagnostic Imaging National University Hospital Singapore Singapore

**Keywords:** cardiac amyloidosis, electrocardiogram, light chain amyloidosis, transthyretin amyloidosis

## Abstract

**Background:**

Cardiac amyloidosis is a protein misfolding disorder involving deposition of amyloid fibril proteins in the heart. The associated fibrosis of the conduction tissue results in conduction abnormalities and arrhythmias. “Classical” electrocardiogram (ECG) findings in cardiac amyloidosis include that of low voltage complexes with increased left ventricular wall thickness on echocardiography. However, this “classical” finding is neither sensitive nor specific. As cardiac amyloidosis is associated with a generally poor prognosis, the need for early recognition of this disease is important given the availability of new treatment options. In this review, we highlight 3 cases of patients with cardiac amyloidosis. Although presenting with typical clinical signs and symptoms, ECG for all 3 patients was not consistent with the classical findings described. They underwent further diagnostic tests which clinched the diagnosis of cardiac amyloidosis, allowing patients to receive targeted treatment. Through the review of the literature, we will highlight the different ECG patterns in patients with different types of cardiac amyloidosis and clinical scenarios, as well as the pitfalls of using ECG to identify the condition. Lastly, we also emphasize the current paradigms in diagnosing cardiac amyloidosis through the non‐invasive methods of echocardiography, cardiac magnetic resonance imaging, and nuclear technetium‐pyrophosphate imaging.

**Conclusions:**

Electrocardiogram is often the first investigation used in evaluating many cardiac disorders, including cardiac amyloidosis. However, classical features of cardiac amyloidosis on ECG are often not present. A keen understanding on the ECG features of cardiac amyloidosis and knowledge of the diagnostic workflow is important to diagnose this condition.

## Graphical abstract

Electrocardiogram (ECG) of a patient with transthyretin amyloid cardiomyopathy, without the classical ECG findings of cardiac amyloidosis. This demonstrates the heterogeneity of ECG findings in cardiac amyloidosis. A high degree of suspicion is often necessary, to further evaluate for possible cardiac amyloidosis.

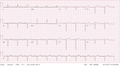

## 1 INTRODUCTION

Cardiac amyloidosis (CA) is a disorder of protein misfolding, with the end result of amyloid fibril deposition in the heart resulting in systolic and diastolic dysfunction. The commonest cause of CA is AL (immunoglobulin light chain amyloid) type, in which amyloid fibrils are derived from monoclonal immunoglobulin light chains (Falk et al., 1997). The other most common amyloidosis affecting the heart would be transthyretin (TTR)‐related amyloidosis (ATTR). ATTR can result either from slow deposition of amyloid fibrils derived from wild‐type (non‐mutant) TTR in the elderly (senile systemic amyloidosis) or when mutations in the *TTR* gene encode variant protein, decreasing the stability of the TTR tetramer and promoting misfolding into amyloid fibrils (Rapezzi et al., 2010). Amyloid deposition in the heart is associated with conduction tissue fibrosis, resulting in conduction abnormalities and arrhythmias (Ridolfi et al., 1977). CA is often under‐diagnosed in the general population and is associated with a variable but generally poor prognosis (Gilstrap et al., 2019; Rapezzi et al., 2009).

Timely diagnosis of CA is challenging because the clinical presentation is similar to other infiltrative cardiomyopathies, storage disorders, and hypertrophic cardiomyopathy. Classical teaching for CA electrocardiography (ECG) includes low voltage complexes (defined by total height of the QRS complex in the limb leads <5 mm and <10 mm in the precordial leads), a pseudoinfarction pattern (pathological Q waves [1/4 of the R amplitude] or QS waves on two consecutive leads in the absence of previous ischemic heart disease, left bundle branch block, or LV wall motion abnormalities), conduction abnormalities, and arrhythmias (Mohty et al., 2013). However, this classical pattern may not always be diagnostic and the clinician often needs to consider other investigations, especially when diagnosing ATTR CA. We present a case series of three patients with ATTR CA, their presenting ECGs, and investigations that they underwent to diagnose their condition.

## 2 CASE 1

**FIGURE 1:**
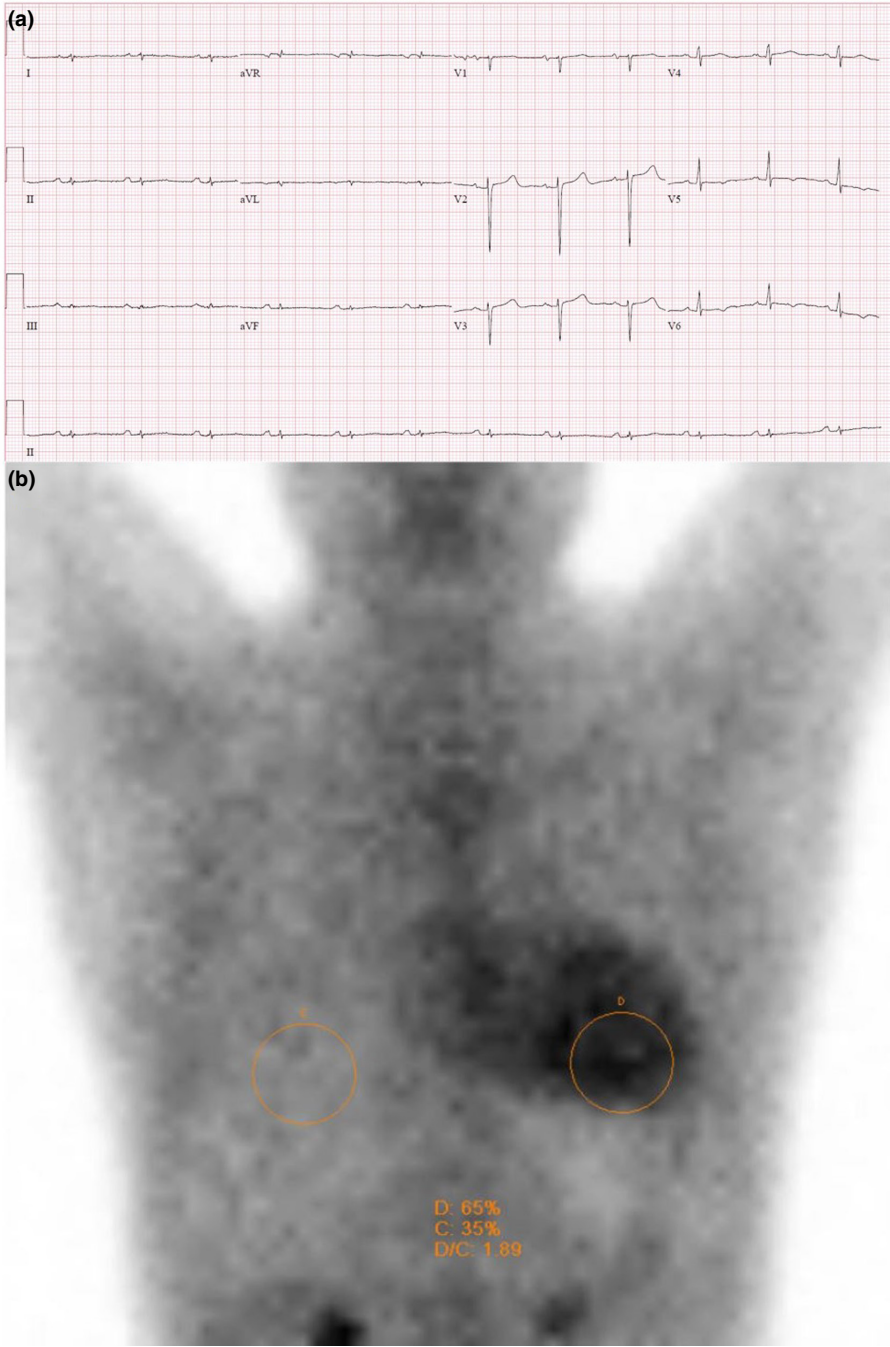
(a) Electrocardiogram of patient showing sinus rhythm with low voltage complexes in the chest leads and pseudoinfarction pattern in leads II and III. (b) Technetium‐pyrophosphate imaging of patient showing diffuse pattern of moderately intense radiotracer uptake seen in the left ventricular myocardium compared with the bony rib cage. Heart‐to‐contralateral lung ratio is 1.89 at 1 h. Semiquantitative interpretation in relation to rib uptake reveals increased myocardial uptake as compared to the rib tracer uptake, score 3. The scintigraphic features are strongly suggestive of transthyretin amyloid cardiomyopathy

A 50‐year‐old gentleman presented with an episode of heart failure. He had complaints of orthopnea and lower limb swelling. He had a family history of heart failure and autonomic neuropathy, present in his mother and maternal uncle. His ECG showed sinus rhythm and QRS size on the limb leads were <5 mm. Average QRS size on the precordial leads was <10 mm. This was in keeping with low voltage QRS complexes. PR interval was 168 ms, QRS duration was 76 ms, and the corrected QT interval (QTc) was 445 ms. There were Q waves in leads II and III. (Figure 1a) These ECG findings were in keeping with the classical findings in CA. Cardiovascular magnetic resonance (CMR) revealed diffuse myocardial thickening with diffuse interstitial fibrosis involving the entire left ventricle, which was suggestive of an infiltrative cardiomyopathy. His myeloma screen was negative for monoclonal light chains, and a bone marrow examination was unremarkable. Nuclear technetium‐pyrophosphate (PYP) imaging showed a Grade III myocardial PYP‐uptake. Heart‐to‐contralateral ratio at 1 hour was 1.89. These findings were diagnostic for ATTR CA. (Figure 1b) He was counseled for genetic testing and was found to have the c.277A>G (p.Ile93Val) pathogenic variant for ATTR. He was started on tafamidis and has been clinically well.

## 3 CASE 2

**FIGURE 2:**
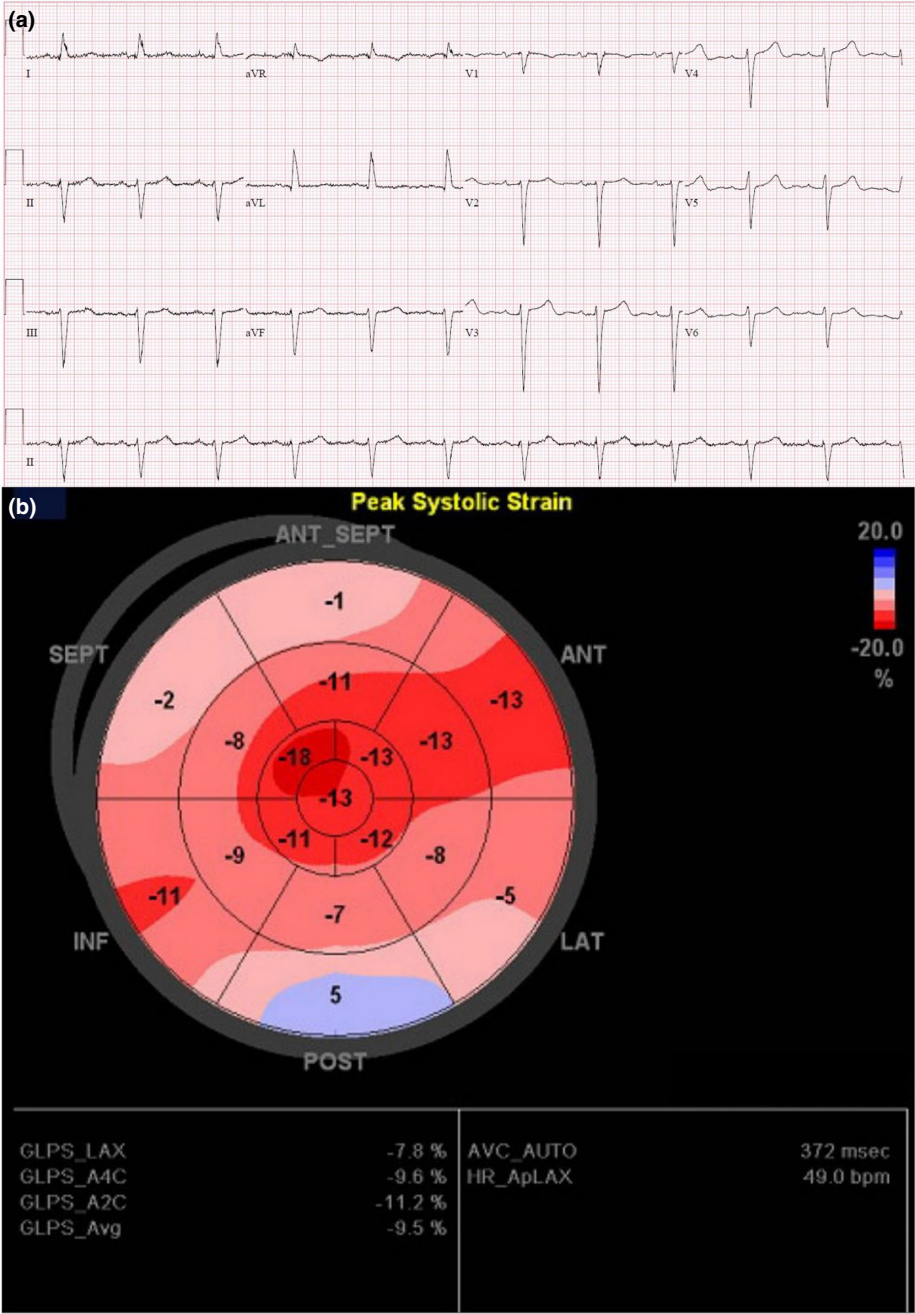
(a) Electrocardiogram of patient showing sinus rhythm, left axis deviation, and incomplete left bundle branch block with large complexes in precordial leads. (b) Left ventricular strain imaging on transthoracic echocardiogram revealing a “cherry‐on‐top” appearance with relative apical sparing

A 68‐year‐old gentleman presented with symptoms of heart failure. He had complaints of exertional shortness of breath, orthopnea, and decreased exercise tolerance with lower limb edema. He has a past medical history of hypertension, which was well‐controlled. This was on a background of positive family history of CA in his elder brother. His ECG showed sinus rhythm, left axis deviation, and incomplete left bundle branch block. Average QRS size on the limb leads was 11 mm, and average QRS size on the precordial leads was 18 mm. PR interval was prolonged at 202 ms. QRS duration was 108 ms, and QTc was 462 mm. (Figure 2a) He did not have low voltage complexes on his ECG. Transthoracic echocardiogram revealed a depressed ejection fraction of 38%, dilated atria, left ventricular hypertrophy (LVH) with global hypokinesia, and apical sparing on strain imaging. (Figure 2b) Coronary angiogram was normal, and myeloma screen was negative for monoclonal light chains. He underwent PYP scan, which showed Grade III myocardial PYP‐uptake. Heart‐to‐contralateral ratio was 2.3, which was diagnostic for ATTR CA. He was treated with doxycycline and ursodeoxycholic acid, and has been clinically well.

## 4 CASE 3

**FIGURE 3:**
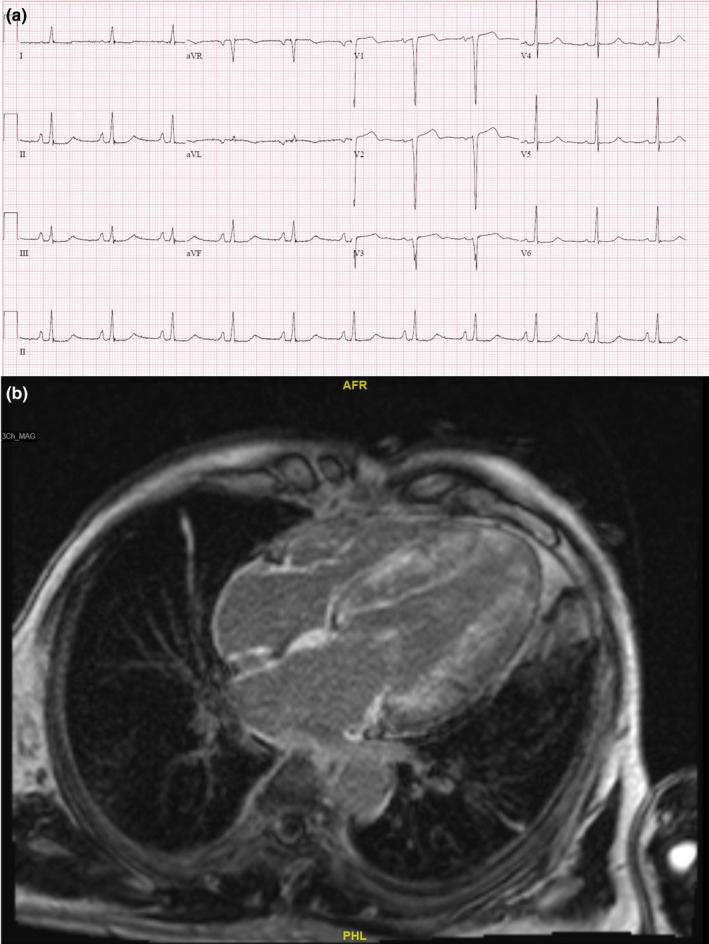
(a) Electrocardiogram of patient showing sinus rhythm, borderline left ventricular hypertrophy, and P pulmonale. (b) Diffuse subendocardial and transmural late gadolinium enhancement (LGE) of the left ventricular myocardium is present. Extensive LGE of right ventricular myocardium, atrial walls, and atrial septum is also noted

A 59‐year‐old gentleman presented with symptoms of bilateral numbness of the extremities and dysautonomia symptoms of postural dizziness. He also had orthopnea and lower limb swelling. He was found to have ATTR and familial amyloid polyneuropathy (FAP) that was diagnosed on sural nerve biopsy. This was on a background of known family history of FAP (father and paternal aunts). His ECG showed sinus rhythm, borderline left LVH, and P pulmonale. Average QRS size on precordial leads was 9 mm, and the average QRS size on the limb leads was 20 mm. PR interval was 164 ms, QRS duration was 98 ms, and QTc was prolonged at 505 ms. (Figure 3a) CMR revealed LVH, mildly impaired left ventricular ejection fraction, and diffuse subendocardial enhancement of bilateral atria and ventricles, which was in keeping with the diagnosis of CA. (Figure 3b). He underwent genetic testing, which came back positive for pathogenic variant Arg54Thr. He is being treated with tafamidis, frusemide, and spironolactone. He remains well on therapy.

### 4.1 ECG pattern differences between AL and ATTR CA

Low voltage complexes on ECG coupled with increased ventricular wall thickness on echocardiography is the classical teaching of CA. This was first described by Carroll et al. (1982) utilizing limb and precordial voltage indices. Nevertheless, low voltage complexes is not a universal finding in CA. There is much variation in the reported prevalence of low voltage complexes in the literature, ranging from 46% to 70%.(Cheng et al., 2011) In another analysis of ECG findings in patients with CA, low limb voltage was found in approximately 35% of patients, while 60% had low Sokolow–Lyon voltage ≤15 mm (Cyrille et al., 2014).

Low voltage complexes appear to be more prevalent in AL CA group than in ATTR CA group (Cappelli et al., 2020). Mussinelli et al. (2013)) showed that low peripheral QRS amplitude (defined as QRS amplitude ≤5 mm [0.5 mV] in each peripheral lead) or low Sokolow–Lyon index (≤15 mm) may represent a useful electrocardiographic clue in the diagnosis of cardiac involvement in a population of patients with AL CA. The corresponding prevalence of AL CA using the low peripheral QRS amplitude criteria was 66.4% and 84.0% when using the low Sokolow–Lyon index criteria. However, when focusing on patients with ATTR CA in the literature, low voltages was only present in about 25% of the study population (Damy et al., 2016; Rapezzi et al., 2009; Rapezzi et al., 2013). A possible reason for this difference in findings is that ATTR CA behaves as a progressive cardiomyopathy (Rapezzi et al., 2009) characterized by slow amyloid deposition within the cardiac chambers and conduction system. AL CA, however, resembles an acute myocarditis with early symptoms onset and rapid disease progression to end‐stage heart failure, despite lesser degrees of infiltration, due to the toxic effects of AL chains (Dungu et al., 2012).

In a study by Dungu et al. (Sperry, Vranian, et al., 2016) looking at a population of patients with ATTR CA, the investigators found that low voltage ECG complexes was an insensitive marker for CA. Low voltage criteria were evident in the limb leads in 15 patients (24.6%) and in the precordial leads in 30 patients (49.2%). However, the investigators did notice that voltage size at initial presentation and during follow‐up was negatively correlated with the duration of symptoms. Thus, a reduction in voltage size is likely to reflect increasing accumulation of amyloid protein over time. This finding of low voltage findings in patients with advanced disease in ATTR CA was also noted in the study by Cyrille et al. (2014) and Sperry, Vranian, et al. (2016) As such, low voltage complexes on ECG is a relatively late finding of ATTR CA and may not be useful for early identification.

Apart from differences in low voltage complexes between AL and ATTR CA, arrhythmias like atrial fibrillation (AF) tend to be more prevalent in ATTR CA, compared with AL CA. Prevalence of AF in ATTR CA was found to be 4 to 6 times more than in AL CA in a study by Cappelli et al. (2020) Another study by Cyrille et al. (2014) also showed similar findings where more than 50% of the patients in the study with ATTR CA had AF as compared to just 6% of the patients with AL CA.

In terms of conduction defects, patients with ATTR CA also present with a higher prevalence of conduction defects including AV block grade 1 or higher and intraventricular delay (Cappelli et al., 2020) This finding was also seen in the study by Cyrille et al. (2014) where more than 50% of the patients with ATTR CA had either primary or secondary AV block as compared to just 26% of patients with AL CA. In both studies (Cappelli et al., 2020; Cyrille et al., 2014), the proportion of ATTR CA patients requiring pacing was much higher than that of those with AL CA.

Pseudoinfarction pattern on ECG is defined by pathological Q waves (1/4 of the R amplitude) or QS waves on 2 consecutive leads in absence of previous ischemic heart disease, left bundle branch block, or left ventricular wall motion abnormalities is a common ECG finding seen in both AL and ATTR CA (González‐López et al., 2017; Murtagh et al., 2005). In patients with AL CA, presence of pseudoinfarction was associated with lower systolic blood pressure, higher N‐terminal pro‐brain natriuretic peptide levels and advanced New York Heart Association (NYHA) heart failure classifications. Patients with pseudoinfarction pattern were also associated with higher prevalence of peripheral low voltage ECG findings, and survival was significantly shorter in the pseudoinfarction group than compared with the group without pseudoinfarction pattern.(Zhao et al., 2016) A possible explanation for presence of pathological Q waves on ECG in patients without overt epicardial coronary obstruction could be due to the deposition of amyloid deposition in the microcirculation and smaller intramyocardial arteries, which is associated with worse outcomes.(Zhao et al., 2016) The study by González‐López et al. (2017) looked at a population of patients with ATTR CA showed that the presence of pseudoinfarction pattern was found to be 60% and was mainly present in the anterior leads. Presence of pseudoinfarction patterns in ATTR CA patients progressively increased with increasing thickness of the interventricular septum and was most common in patients with the thickest hearts (Damy et al., 2016).

### 4.2 QRS complex size relative to degree of LVH

The study by Carroll et al. (1982) showed that voltage–mass ratio was a good indicator for CA. Voltage–mass ratio is defined as Sokolow–Lyon index divided by the cross‐sectional area of the left ventricular wall and a ratio of <1.5 was suggested to be indicative of CA. This has since led to the recommendation to consider CA in patients with LVH on echocardiogram but yet have small QRS complexes.(Ruberg et al., 2019) This is especially the case with regards to patients with AL CA where many studies have shown a high prevalence of low voltage–mass ratio.(Murtagh et al., 2005; Sperry, Vranian, et al., 2016; Zhao et al., 2016) However, when comparing AL CA to ATTR CA, the mean voltage‐to‐mass ratio was higher in patients in ATTR CA. The study by Rapezzi et al. (2009)) looked at the difference in voltage–mass ratio between AL CA and ATTR CA. ATTR CA patients less often displayed low voltage‐to‐mass ratio (1.1 ± 0.5 in ATTR CA versus 0.9 ± 0.5; *p* < .0001in AL CA) (Rapezzi et al., 2009). The findings of a higher mean voltage–mass ratio in ATTR CA patients is in keeping with the lower prevalence of low QRS complexes in patients with ATTR CA.

Another consideration is the use of the total QRS score (defined by the sum of all QRS voltages in all leads) divided by the left ventricular mass index. This finding was found to be rather sensitive (81%–87%) and specific (79%–82%) in the differentiation of CA to other conditions that result in LVH like hypertensive heart disease and hypertrophic cardiomyopathy (Quarta et al., 2012). This finding was also seen in another study by Perlini et al., which showed a strong association of a depressed total QRS score to left ventricular mass index in patients with AL CA with an area under the receiver operating characteristic curve for the detection of AL CA of 0.96 (95% CI, 0.93–0.98) (Perlini et al., 2010).

### 4.3 ECG in presence of background chronic pressure overload

It would be interesting to study the ECG patterns of patients with CA and have a concomitant background of chronic pressure overload. There is growing recognition of an increasing prevalence of patients with ATTR CA in patients diagnosed with aortic stenosis (AS). Some studies report a prevalence of 25% (Cavalcante et al., 2017) in patients with AS, and this is especially in older patients and is also associated with worse outcomes. In the study by Castano et al. (2017)) patients with AS diagnosed with ATTR CA compared with those with isolated AS had longer QRS duration (127 vs.110 ms, *p* = .017) and higher prevalence of right bundle branch block (37.5% vs. 15.8%, *p* = 0.023). The most common arrhythmia in a reported series was AF, present in 41.7%–67% of patients with concomitant AS and ATTR CA (Castano et al., 2017; Cavalcante et al., 2017; Sperry, Jones, et al., 2016). Even though AF is a frequent finding in patients with AS, when studying patients with both ATTR CA and AS, the prevalence of atrial arrhythmias was significantly higher: 67% in the ATTR CA group vs. 20.2% in the isolated AS group, *p* = .006.(Cavalcante et al., 2017) Another study showed that voltage–mass ratio was lower in patients with patients with CA and AS than compared with isolated AS (0.9 × 10–2 mV/g/m2 (0.6–1.6) vs. 1.6 × 10–2 mV/g/m2 (1.1–2.3); *p* = .001) too (Nitsche et al., 2020).

The classical ECG findings of patients with hypertension include LVH pattern. Criteria to establish LVH in the literature are numerous, and the Cornell criteria (Okin et al., 1995) and the Sokolow and Lyon (1949) criteria are common criteria used to define LVH on ECG. CA leads to increased left ventricular wall thickness due to the abnormal deposition of amyloid fibrils in the ventricular wall. However, the presence of LVH on ECG in patients with CA is low, especially in patients with AL CA as they have a higher prevalence of low voltage complexes. With regard to the ATTR CA population, majority of the patients do not have LVH pattern on their ECGs but some studies do show that there is a small proportion of patients (12%–16%) who may have LVH pattern.(Dungu et al., 2012; González‐López et al., 2017; Maurer et al., 2016)

### 4.4 Additional investigations to aid in diagnosis of CA

Electrocardiogram is not a sensitive modality for diagnosing CA and its subtypes. Other non‐invasive imaging modalities are available to aid clinicians in diagnosing ATTR CA.

Echocardiography plays a major role in non‐invasive diagnosis of cardiac amyloidosis due to its ability to assess the structure and function of the heart. As described in our case series, the presence of apical sparing on strain imaging is a useful tool to aid in the diagnosis of CA (Phelan et al., 2012). Strain imaging has the ability to refine the non‐invasive recognition of cardiac amyloidosis by quantitating longitudinal systolic function. Patients with CA demonstrate a typical pattern of distribution in which basal LV segments are severely impaired while apical segments are relatively spared (“Cherry‐on‐the‐top” sign on longitudinal strain bullseye map). Therefore, strain imaging should be performed in patients with unexplained LVH.

Cardiovascular magnetic resonance has a central role in the non‐invasive diagnosis of CA due to its ability to provide tissue characterization in addition to high‐resolution morphologic and functional assessment (Martinez‐Naharro et al., 2017). The common findings on CMR in patients with CA are that of global subendocardial and transmural late gadolinium enhancement (LGE). Both patterns are present in AL and ATTR CA, but to different extents, with subendocardial LGE being more prevalent in AL and transmural LGE more prevalent in ATTR CA (Fontana et al., 2015). Other findings of CA include nulling of myocardium before or at the same inversion time as the blood pool, LGE of left atrial wall, and extensive extracellular volume expansion which are combined with structural findings of increased wall thickness and myocardial mass. CMR, however, is unable to definitively distinguish AL from ATTR CA and should be combined with electrocardiographic, clinical, biomarker, and other imaging findings to maximize diagnostic accuracy (Dorbala et al., 2021).

Radionuclide imaging plays a unique role in the non‐invasive diagnosis of CA. A variety of ^99m^Tc‐labeled diphosphonate and PYP (bone‐avid) compounds diagnose ATTR CA with high sensitivity and specificity (Gillmore et al., 2016). With a negative myeloma screen (absence of monoclonal protein using urine and serum, with serum‐free light chain assay and immunofixation electrophoresis) and a positive finding of grade 2 or 3, myocardial radiotracer uptake on bone scintigraphy allows a positive predictive value for ATTR CA of 100% (95% CI, 98.0%–100%) and thus obviating the need for an endomyocardial biopsy (Gillmore et al., 2016). The study by Bokhari et al. (2013) also showed that the semiquantitative heart‐to‐contralateral ratio of ≥1.5 is also another accurate marker to diagnose ATTR CA if AL has been excluded through a negative myeloma screen.

## 5 CONCLUSION

With the recent introduction of novel strategies to inhibit amyloid fibril formation (such as tafamidis for ATTR CA), the need for early diagnosis of ATTR CA through non‐invasive methods is ever more important. Using our case examples, we showed that using “classical finding” of low ECG voltage complexes as a diagnostic tool for CA is not ideal. Large‐voltage complexes should not prevent further investigation for infiltrative cardiomyopathy either. If clinical suspicion for CA is high, there are other non‐invasive methods such as strain imaging on echocardiography, CMR and nuclear scintigraphy that can aid us in earlier detection of this sinister disease.

## CONFLICT OF INTEREST

There is no conflict of interest for all authors.

## AUTHORS' CONTRIBUTIONS

Perryn Lin Fei Ng contributed to the conception of article, acquisition of data, data analysis, data interpretation, and manuscript drafting and revisions. Weiqin Lin contributed to the acquisition of data, data analysis, data interpretation, as well as manuscript drafting and revisions. Yoke Ching Lim, Lauren Kay Mance Evangelista, Raymond Ching Chiew Wong, Ping Chai, Ching Hui Sia, Hoi Yin Loi and Tiong Cheng Yeo contributed to the data interpretation as well as manuscript drafting and revisions.

### ETHICS APPROVAL AND CONSENT TO PARTICIPATE

Ethics approval is waived as per institutional guidelines.

### CONSENT FOR PUBLICATION

Written informed consent was obtained from the patient for publication of this case report and any accompanying images. A copy of the written consent is available for review by the Editor of this journal.

### DATA AVAILABILITY STATMENT

Deidentified data can be made available by the corresponding author, upon reasonable request.

## Data Availability

The data that support the findings of this study are available on request from the corresponding author. The data are not publicly available due to privacy or ethical restrictions.
